# Embedding Dementia Care Navigation into Primary Care

**DOI:** 10.1093/geroni/igaf122.608

**Published:** 2025-12-31

**Authors:** Janice Knebl, Jennifer Severance, Sara Murphy

**Affiliations:** University of North Texas Health Science Center, Fort Worth, Texas, United States; University of North Texas Health Science Center - Fort Worth, TX, Fort Worth, Texas, United States; University of North Texas Health Science Center, Fort Worth, Texas, United States

## Abstract

People living with dementia often have multiple chronic conditions and receive fragmented care, leading to high rates of hospitalization and emergency visits. Dementia care navigators provide person-centered resources, education, and support to help caregivers understand and access their options and have been found to improve health outcomes for people living with dementia. However, the adoption of dementia care navigator services by health systems is limited due to a variety of factors, including lack of defined delivery models and the absence of a payment structure for primary care. This presentation describes a primary care clinic’s experience as one of two healthcare providers on the experienced program track in Texas approved by the Centers for Medicare and Medicaid Services (CMS) to offer the Guiding an Improved Dementia Experience (GUIDE) Model designed to improve the quality of life for people living with dementia and those in their lives who give them support. The GUIDE Model pays for the coordination of services, education about dementia, and the support people living with dementia often need to remain in their homes safely and delay nursing home care as long as appropriate. The clinical team also received a grant from the state to assess emerging and unmet needs of persons with dementia and their caregivers and to strengthen community-clinical linkages through dementia resources specialists. Early results and learnings reveal challenges and opportunities of implementing dementia care navigator services and the need for comprehensive and coordinated approaches to dementia care addressing needs at the individual and community levels.

